# Using observational facial descriptors to infer pain in persons with and without dementia

**DOI:** 10.1186/s12877-018-0773-8

**Published:** 2018-04-11

**Authors:** Stefan Lautenbacher, Anna Lena Walz, Miriam Kunz

**Affiliations:** 1 Physiological Psychology, University of Bamberg, Markuspl. 3, 96045 Bamberg, Germany; 2 Department of General Practice and Elderly Care Medicine, University Medical Center Groningen, University of Groningen, Groningen, The Netherlands

**Keywords:** Pain, Pain assessment, Dementia, facial expression, Pain behavior, Observer scales

## Abstract

**Background:**

For patients with advanced dementia, pain diagnosis and assessment requires observations of pain-indicative behavior by others. One type of behavior that has been shown to be a promising candidate is the facial response to pain. To further test how pain-indicative facial responses are, we investigated the predictive power of observational facial descriptors to (i) predict the self-report of pain and (ii) to differentiate between non-painful and painful conditions. In addition, the expertise of the observers (nurses vs. healthy controls) and the cognitive status of the observed (dementia vs. cognitively healthy) were considered.

**Methods:**

Overall 62 participants (32 nurses and 30 control subjects) watched 40 video-clips, showing facial expressions of older individuals with and without dementia during non-painful and painful pressure stimulation. After each clip, participants were asked to rate the videos using commonly used facial descriptors of pain and also to provide global pain estimate ratings of how much pain the observed individual might have experienced.

**Results:**

Out of the 12 facial descriptors used, only 7 were able to differentiate between non-painful and painful conditions. Moreover, participants were better in predicting the pain self-report of the observed individuals when using facial descriptors than when using global pain estimates. Especially, the anatomically-orienting descriptors (e.g. opened mouth, narrowing eyes) showed greatest predictive power. Results were not affected by pain-expertise of the observers (nurses vs. control subjects) or diagnostic status of the observed (patients with dementia vs. cognitively unimpaired subjects).

**Conclusions:**

The fine-grained and specific observation of facial responses to acute pain appeared to provide valid indication of pain that is not compromised when patients with dementia are observed. The regular professional training does not put nurses at advantage to detect pain via facial responses.

## Background

When dementia progresses to moderate or severe stages, the abilities of introspection, self-monitoring and self-report are so severely compromised (due to the cognitive decline) that pain detection and assessment are substantially hampered [1, 2]. The subjective experience of pain is no longer accessible via self-report. Therefore, observable behaviors gain more and more importance as remaining indicators of pain. There is wide agreement that the behaviors in three domains are especially pain indicative, namely facial responses, body movement/posture, and vocalization [3]. Basic knowledge and methodology of assessment are certainly most advanced for the facial responses accompanying the experience of pain [4–6]. One of the most prominent methods to assess and analyze such facial responses is the Facial Action Coding System (FACS) [7]. FACS and related coding systems allow for a very precise coding of neuromuscular activity in the face based on video recordings of the patients. FACS has proven high validity and reliability, both in healthy individuals as well as in patients with mild to moderate stages of dementia [8–11]. Nevertheless, it is too time and staff consuming for clinical use. There will be video- and computer-based systems available in the future, which might assist or substitute the FACS coder and allow for online coding of facial expressions in clinical care settings [12]. For now, however, the best alternative for clinical use seems to be the behavioral observation of facial responses [3, 13].

For that purpose, validated behavioral observation criteria are necessary, in order train health care professionals and guide the pain evaluation of patients. Although all established observational scales for the assessment of pain in dementia include items relating to facial responses, these items have rarely been validated for targeting the criterion “pain”. One attempt has been made by Sheu et al. [6], using videotapes of elderly individuals undergoing painful procedures. Five coders rated the facial responses by use of common observational pain scales (only facial items). Sheu et al. found that those scales including more anatomically descriptive items performed much better as regards reliability and validity (correlation with self-report and FACS) than those with more interpreting items. Following a similar approach as Sheu et al. [6], we are aiming to develop an observer scale that includes the best pain-indicative facial items. The so-called PAIC^1^-FACE-SCALE is based on an item pool, which became available by extracting items from the most widely used observer scales for pain assessment in dementia [14]. The first application of the PAIC-FACE-SCALE (research version, 13 items) took place in nursing homes where caregivers were asked to use the scale and in addition provide overall pain estimation for the observed residents, mainly patients with dementia [15]. The caregivers mostly observed patients during situations with a high likelihood of the occurrence of pain (e.g. “transfer to wheel-chair”). We found that caregivers based their overall pain estimation on only part of the facial descriptor items. In agreement with the findings of Sheu et al. [6], the anatomically orientating items (e.g. “narrowing eyes”) proved to be the best predictors, followed by items indicating facial expressions of emotional states (e.g. “looking tense”).

This study gave us first valuable insights into the use of facial descriptors in everyday pain evaluation by caregivers in nursing homes. However, it neither informs us of whether and how the facial items relate to the gold standard in pain evaluation, namely the self-report of the observed persons; nor how the facial items correspond with objective measures of pain, i.e. application of non-noxious vs. noxious stimuli. For that purpose, we conducted the present study where we applied a similar design as Sheu et al. [6] and used video recordings of persons, who had been stimulated with non-noxious or noxious pressures and who had been asked for self-reporting their pain. These videos were shown to the study participants, who were instructed to rate the facial responses by utilizing the PAIC-FACE-SCALE. This approach allowed us relating the observed facial responses to the self-report of pain and to the responses to non-noxious vs. noxious stimuli and thus, answer the question of which of the facial descriptors are most pain-indicative.

Two potential influences on the observational evaluations in these situations were studied further: (i) Experience with and prior training in pain management may affect the observational performance [16–18]. For example, longstanding experience in pain management has been associated with greater underestimation of pain in others [16]. To test for such potential biases, we compared nurses with longstanding work experience and laypersons. (ii) Although patients with dementia have been found to express pain similarly as cognitively healthy individuals [8–11], we included video recordings of both cognitively healthy and impaired individuals of advanced age, to further put the assumption of potential group differences to test.

## Methods

### Participants

The participants were 32 nurses (hospital nurses and elderly care nurses) and 30 control participants (no paramedical professions, mainly secretaries and administrative officers) that were matched for age, gender and education (see Table 1). Nurses were recruited from a large local hospital (Sozialstiftung Bamberg (45%) with various specialized units) as well as from local nursing homes (55%). We only included nurses who had at least 3 years of work experience and were mainly working with elderly individuals. On overage, nurses had a work experience of more than 10 years and reported that 54% of their patients were suffering from dementia. In addition, nurses reported that 1/3 of their patients were suffering from pain. Control participants were recruited via advertisements posted in the university buildings (University of Bamberg) and posted in local newspapers. We only included control participants who were not caring for a patient with chronic pain or a patient with dementia. Exclusion criteria for both groups of participants were acute or chronic pain, mental disorders in the last ten years, somatic diseases with likely affection of the pain system, self-reported impaired vision, disorder of attention and prosopagnosia. These criteria were recorded by use of an anamnesis questionnaire. The study protocol was approved by the ethics committee of the Otto-Friedrich University of Bamberg. All participants gave written informed consent. All individuals were paid for participation (20 €).

**Table 1:** Descriptive data of the two samples studied

		Nurses	*Controls*
*N*		32	30
Age (in years)		40.3 (11.3)	40.0 (12.3)
Sex (male/female)		7/25	3/27
Education	lower secondary school (Hauptschule)	7	4
	Intermediate secondary school (Realschule)	18	22
	Higher education entrance qualification (Abitur) (finished or enrolled)	5	4
	University degree	2	0

### Video material

The video segments, which were presented to the nurses and control participants in the present study, were recorded in earlier studies on facial expressions of pain (for more detail on the experimental protocol please see description in Kunz et al. [8, 10]). Two groups of individuals were shown in these videos, namely older individuals without cognitive impairment (above 65 years (mean MMSE score = 29.1.0, SD: 0.6; range 28–30)) and older individuals with mild to moderate forms of dementia (above 65 years (mean MMSE score = 17.0, SD: 4.9; range 10–21)). For more details on the video material used, please see our previous publications [19, 20]. In short, the face of the individuals was videotaped while they received pressure stimulation of non-painful (2 kg) and painful (5 kg) intensities. After each stimulation, the videotaped individuals were asked to give a self-report rating (“no pain”, “slight pain”, “moderate pain”, “strong pain”, “very strong pain”, “unbearably strong pain”; verbal rating scale, VRS) and all video clips were also analyzed using the Facial Action Coding System [7]. For each of the two videotaped groups (healthy elderly, patients with dementia), the video material of 10 individuals (♀ = 5; ♂ = 5) were randomly selected [19, 20]. Altogether, 40 five-seconds video segments (2 intensities x (10 healthy elderly + 10 patients with dementia)) were presented to each observer in the present study in a randomized order. All videotaped individuals had provided written informed consent that their video recordings can be used in future research studies of our research group (the study protocol had been approved by the ethical committee of the University of Marburg).

### Observer judgements of the video material

Participants were asked (i) to rate all videos using twelve facial descriptors of pain and (ii) to provide two overall estimates of the pain that the individual in the video might have experienced. These two types of rating scales are described in detail in the following.

### Facial descriptors of the PAIC-FACE-SCALE

We selected facial descriptor items from established observational pain assessment tools for people with dementia in several steps which are described in detail elsewhere [14, 15]. In brief, out of well-established pain assessment tools (The ABBEY Pain Scale [21], ADD [22, 23], CNPI [24, 25], DS-DAT [26, 27], DOLOPLUS-2 [28], EPCA-2 [29], MOBID-2 Pain Scale [30], NOPPAIN [31], PACSLAC [32], PAINAD [33], PADE [34], and PAINE [35]), all items relating to facial expressions were extracted. After removing largely or completely synonymic items, we further reduced the number of items by selecting the most promising facial descriptors based on published research on the facial expressions of pain [36–38], on the frequency of occurrence in existing pain assessment tools as well as on the opinion of pain experts (EU-grant: COST Action TD 1005; 11) as regards their clinical utility. This resulted in a final pool of 13 facial descriptors [15]. For the present study, we had to exclude one of the 13 selected facial descriptor, namely “pale face”, given that our videos were b/w and, thus, not suitable to judge paleness of the face. Thus, we only used 12 facial descriptors (see Table 2) in the present study. Facial descriptors were scored on a 4-point category scale, describing how well the descriptor item applied coincided with the facial response of the videotaped individual (not at all - slight degree – moderate degree – great degree). There was also the option to select “not applicable/not scoreable” as an answer.

**Table 2:** Step 1 - Selecting pain-indicative facial descriptors that are (a) observed in more than half (> 50%) of the pain videos (5 kg) and (b) that can differentiate between no-pain (non-noxious 2 kg stimulatus) and pain (noxious 5 kg stimulus) (effect size > 0.80)

	A. Frequency with which the facial descriptors were used; when judging facial expressions to 5 kg pain (in percentage (%))			B. Ability of a facial descriptor to differentiate between facial expressions to 2 and 5 kg (effect size, Cohen’s d)			Selected for further analyses^1^
	All videos	Healthy elderly	Patients with dementia	All videos	Healthy elderly	Patients with dementia	
Frowning	**80%**	**81%**	**79%**	**4.61**	**2.56**	**3.38**	x
Narrowing eyes	**61%**	**56%**	**66%**	**4.79**	**2.33**	**4.61**	x
Closing eyes	26%	18%	34%	**2.29**	−1.25	**4.50**	
Raising upper lip	**54%**	**53%**	**55%**	**5.09**	**3.34**	**4.43**	x
Opened mouth	**53%**	**50%**	**56%**	**5.37**	**2.71**	**5.26**	x
Tightened lips	**58%**	**55%**	**61%**	**1.10**	**0.86**	**0.88**	x
Empty gaze	**57%**	**55%**	**59%**	−2.27	−1.13	−1.61	
Seeming disinterested	47%	48%	46%	−2.55	−1.30	−1.73	
Teary eyes	32%	28%	37%	**0.94**	0.13	**1.00**	
Looking tense	**83%**	**80%**	**86%**	**4.03**	**1.79**	**3.54**	x
Looking sad	**76%**	**72%**	**81%**	−0.28	−0.93	0.33	
Looking frightened	**70%**	**65%**	**74%**	**2.86**	**1.38**	**2.09**	x

Values are given separately for all videotaped individuals (healthy older individuals and patients with dementia), as well as for each videotaped group separately

^1^ *Only those facial descriptors are selected for further analyses that meet both selection criteria (A and B) for all videos and for the videotaped groups of healthy controls and patients with dementia, separately*

### Overall estimates of the pain

Participants were asked to give in addition two overall estimates of pain for each video. Firstly, a rating for pained expression (“How intense is the pained expression?”) and secondly, a rating for pain intensity (“How intense is the pain the individual is experiencing?”) were presented. These two overall pain estimate ratings had to be scored on two 4-point category scales (no pain (no pained expression)- slight – moderate – great).

### Experimental protocol

The presentation of the video segments and the assessment of the observer ratings were made possible by the use of a laptop (screen width of 15.4 in.). Testing took place in a quiet room either in our laboratory at the University of Bamberg or in the clinical center / nursing homes. The session lasted for approximately 90 min and was divided into 2 blocks. In each block subjects watched and rated 20 video segments. There was a 15 min break between blocks to allow for a short recreation period. Participants were told that the individuals in the videos were recorded while they were experiencing different levels of pain and non-painful sensations. Participants were instructed that they should look at each video carefully and that after each video they were going to be asked to judge what they observed in the video.

**Fig. 1:**
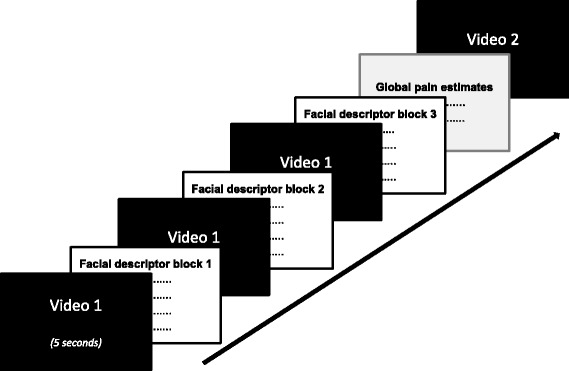
Experimental design of the video presentations and the assessment of the observer ratings

After each video, participants were asked to score their observations using the 12 facial descriptor items and the 2 global pain estimates. Given that it is too demanding to focus on 12 different facial descriptors at the same time, the descriptors were split up into three facial descriptor rating blocks. Therefore, each video was consecutively presented three times, followed each time by the request to rate 4 facial descriptor items (see Fig. 1). The order, in which facial descriptors were presented, was randomized across participants but was hold stable within one participant. Following the last facial descriptor rating block, participants were asked to complete the 2 overall pain estimate ratings (see Fig. 1). Each rating block was terminated when the participants had scored all items presented (by mouse-click). Before starting the testing procedure, participants were familiarized with the rating procedure using two training videos. Thus, after completion of the training phase, participants were already familiar with the order in which they had to score the facial descriptors and the overall pain estimates.

### Statistics

To investigate which of the facial descriptors are best suitable to assess pain, we used the following steps:

(i) *Step 1: Ability to differentiate between non-painful and painful conditions*
  In step 1, we wanted to investigate, which of the facial descriptors can differentiate between non-painful and painful conditions. In order to answer this question, we calculated the frequency with which each facial descriptor item was scored during pain (videos showing facial responses to the noxious 5 kg pressure stimulus). A pain-indicative item should be scored in at least 50% of the cases. Moreover, we calculated whether a facial descriptor item was scored higher during pain (facial response to the noxious 5 kg stimulus) compared to no pain (non-noxious 2 kg stimulus) videos. Only those facial descriptors that were chosen in at least half of the pain video segments and, in addition, scored considerably higher during pain vs. no-pain videos (effect size (Cohen’s d) > 0.80, large effect) were selected as pain-indicative facial descriptors. All calculations were computed once including videos of all participants together as well as separately for the videos of healthy elderly and of patients with dementia.
(ii) *Step 2: Ability to predict differences in self-reported pain intensity*
  In step 2, we wanted to investigate which of the facial descriptors are not only capable of differentiating between non-painful and painful conditions (step 1) but can also predict self-reported pain intensity of the observed person (step 2). Thus, we selected the facial descriptors that proved pain-indicative in step 1 (pain-indicative facial descriptors). These pain-indicative facial descriptors were entered as predictor variables in linear regression analyses, with the self-reported pain intensity ratings of the videotaped individuals as criterion variable. In order to determine best predictors, we computed beta weights, zero-order correlation and a product measure of these two. The product measure has been supposed to be a good measure for ranking the predictors according to their predictive power [39].
  Regression analyses were only conducted for videos showing facial responses to painful stimulation (5 kg). Moreover, regression analyses were conducted, analyzing all videos and observers at once as well as separately for nurses and controls as observers as well as separately for videos of healthy elderly and patients with dementia, resulting in 5 regression analyses.
(iii) *Step 3: Additional gain in diagnostic accuracy when using facial descriptors compared to only global pain estimates*
  In step 3, we wanted to investigate whether it is really preferable to use specific facial descriptors when inferring pain in others or whether a simple global pain estimate (e.g. asking observers to rate: how intense is the pain the individual is experiencing?) might already be sufficient.

Therefore, stepwise linear regression analyses were conducted, entering the global pain estimates (“pain intensity estimate” and “pained expression estimate”, respectively) in the first predictor block and the pain-indicative facial descriptors in the second block. A significant gain of explained variance (change in r^2^) in step 2 would prove the necessity to use facial descriptors. Criterion was always the self-report of pain of the videotaped individual.

The α-level was set to 0.05 throughout and analyses were conducted using SPSS 20.

## Results

### **Step 1:***(i) Ability to differentiate between pain and non-painful conditions*

As can be seen in Table 2 (A.), all of the 12 selected facial descriptors were chosen for at least 18% of the pain videos (showing facial responses to noxious 5 kg pressure stimulation). With the exception of three facial descriptors (“closing eyes”, “seeming disinterested”, “teary eyes”), the remaining facial descriptors were scored – as requested - in more than half of the pain videos. Interestingly, the frequency, with which facial descriptors were chosen, was very comparable between videos of healthy elderly and patients with dementia (see Table 2 (A.)).

When computing effect sizes (see Table 2 (B.)) to investigate which of the facial descriptors scored substantially higher during pain (videos showing responses to noxious 5 kg pressure stimuli) compared to non-pain (videos showing responses to non-noxious 2 kg pressure stimuli), we found that 7 out of the 12 facial descriptors were able to clearly differentiate between pain and non-pain across videos of healthy elderly and patients with dementia. Since we were looking for strong effects that are of clinical relevance, we chose a Cohen’s d score of > 0.80 (indicating strong effect size) as the cut-off. As can be seen in Table 2 (B.), effect sizes for the differences between non-pain and pain were often meaningfully higher (difference between Cohen’s d effect sizes > 0.5) for patients with dementia compared to healthy controls, indicating that the observer participants were better able to discriminate pain versus non-pain expressions (using the facial descriptors) when watching patients with dementia. The 7 facial descriptors which showed to be pain-indicative in step 1 (see Table 2, last column) were selected for further analyses (step 2 and 3).

### **Step 2:***(ii) Ability to predict differences in self-reported pain intensity*

We conducted regression analyses to test which of the pain-indicative facial descriptors (selection in step 1) can best predict the self-reported pain of the videotaped individuals. When conducting the regression across all videos and all participants (left side of Table 3), we found that the 7 selected facial descriptors were indeed able to predict the self-reported pain intensity of the videotaped individuals. Overall facial descriptors were able to significantly explain 9% of variance in self-reported pain intensity ratings. As indicated by the product measure, the facial descriptors “opened mouth”, “raising upper lip”, “frowning” and “narrowing eyes” were the most important predictors; whereas “looking frightened”, “looking tense” and “tightened lips” were of less importance in the regression equation.

**Table 3:** Step 2 - Which facial descriptors can best predict the self-report of pain (criterion: self-report of pain)

Videos:	All videos showing facial responses to noxious 5 kg pressure stimuli			All videos showing facial responses to noxious 5 kg pressure stimuli		Healthy elderly (“5 kg”)	Patients with dementia (“5 kg”)
Participant group:	All participants			Nurses	Controls	All participants	
Overall model fit:	R^2^ = 0.093 (*p* < .001)			R^2^ = 0.091 (*p* < .001)	R^2^ = 0.109 (*p* < .001)	R^2^ = 0.160 (*p* < .001)	R^2^ = 0.116 (*p* < .001)
	Beta weight	Zero-order r	Product measure*	Product measure	Product measure	Product measure	Product measure
Opened mouth	.332	.212	.070	*.054*	*.090*	*.109*	*.047*
Raising upper lip	.177	.082	.015	*.019*	*.009*	*.001*	*.056*
Frowning	.084	.064	.006	*.014*	*.001*	*.044*	*.003*
Narrowing eyes	.065	.058	.004	*.011*	*.001*	*.009*	*.003*
Looking frightened	.024	.042	.001	*<.001*	*.004*	*<.001*	*.002*
Looking tense	.005	.018	<.001	*<.001*	*.002*	*.007*	*.006*
Tightened lips	.069	−.021	< −.001	*−.001*	*.006*	*−.008*	*<.001*

Values are given separately for the whole sample and the whole videos presented, as well as separately for the videotaped groups of healthy older individuals and patients with dementia and separately for the two subject groups (nurses, controls)

*The product measure is a combination of beta weights and zero order correlations and uniquely reflects both direct and total effects of each predictor variable

When conducting regression analyses separately for nurses and controls (see middle columns of Table 3), very similar finding were found. Explained variance was again 9–10% and the facial descriptors “opened mouth” and “raising upper lip” proved again to be the most important predictors. Thus, the predictive value of the facial descriptors was not dependent on the professional pain expertise of the observer.

When conducting regression analyses separately for videos showing patients with dementia and healthy elderly individuals (see right columns of Table 3), we found that facial descriptors were able to explain 16% of the variance in self-reported pain intensity in the healthy elderly, whereas only 12% explained variance was found for patients with dementia. Moreover, depending on the videotaped group, other facial descriptors proved to be most important. Whereas the facial descriptor “opened mouth” was always among the most relevant predictors, “frowning” proved to be very important for predicting self-report of healthy elderly, whereas in patients with dementia “raising upper lip” was the most important predictor.

### **Step 3:***(iii) Additional gain in diagnostic accuracy when using facial descriptors compared to only global pain estimates*

In the last step, we used step-wise regression analyses to investigate which additional gain in diagnostic accuracy can be derived from using facial descriptors in addition to global pain estimates. As can be seen in Table 4, the global pain estimates (pain intensity estimate and pained expression estimate) were not able to predict self-reported pain intensity of the videotaped individuals. Entering the 7 pain-indicative facial descriptors in the second block led to a significant increase of explained variance (9%) compared to the two global pain estimates. Thus, substantial additional explanatory power gain can be derived from using facial descriptors compared to global pain estimates.

**Table 4:** Step 3 - Step-wise regression analyses to assess the predictive gain of using facial descriptors to infer pain in others compared to (a) an overall pain intensity estimate rating or (b) an overall pained expression estimate

Blocks	Predictors	*r*	R^2^	Change in R^2^	F value of the change	Significance of the change (*p*-value)
a						
1	Pain intensity estimate (“How intense is the pain the individual is experiencing?”)	.075	.006			
2	Opened mouth/Raising upper lip/Frowning/Narrowing eyes/Looking frightened/Looking tense/Tightened lips	.311	.097	.091	17.06	<.001
b						
1	Pained expression estimate (“How intense is the pained expression?”)	.054	.003			
2	Opened mouth/Raising upper lip/Frowning/Narrowing eyes/Looking frightened/Looking tense/Tightened lips	.309	.095	.092	17.10	<.001

Criterion: self-report of pain of the videotaped individuals

### Association between (anatomically-based) facial descriptors and FACS analyses

The facial descriptors that proved to be most pain indicative (step 1) as well as pain-related enough to predict self-reported pain intensity (step 2) were interestingly mainly anatomically-orientating items that have their counterparts in the Action Units (AUs) of the Facial Action Coding System (FACS) [7]. Thus, correlations were computed between facial descriptors (scored by the observer participants) and their corresponding Action Units coded by a trained FACS coder. The results are displayed in Table 5. As can be seen, the strongest correlations were obtained between the facial descriptor “opened mouth” and the corresponding AUs 25_26_27 (moderate strength). The facial descriptors “narrowing eyes” and “raising upper lip” showed mostly weak associations with their corresponding AUs (AUs 6_7 and AUs 9_10). Only the facial descriptor “frowning” showed no noteworthy association with its corresponding AU 4. These correlations were not affected by the pain expertise of the observer, given that nurses and controls yielded similar findings. A tendency for stronger associations was found for the videos of patients with dementia.

**Table 5:** Correlations (r-values) between facial descriptors (scored by the participants) and their corresponding Action Units (fine-grained FACS analysis)

Correlation between:	All videos	All videos		Healthy elderly	Patients with dementia
	All participants	Nurses	Controls	all participants	
Frowning x AU 4	.071	.075	.068	−.244 ***	.220***
Narrowing eyes x AU 6_7	.297***	.295***	.299***	.004	.400***
Raising upper lip x AU 9_10	.336***	.342***	.330***	.449***	.250***
Opened mouth x AU 25_26_27	.535***	.500***	.571***	.530***	.541***

Values are given separately for the whole sample and the whole videos presented, as well as separately for the videotaped groups of healthy older individuals and patients with dementia and separately for the two subject groups (nurses, controls)

***p < .001

## Discussion

The major findings of the present study were that the use of facial descriptor items (i) helped observers to distinguish conditions in which people experienced pain due to noxious stimulation from non-painful conditions and (ii) allowed for predicting the self-report, but only to a small degree. These findings will be discussed first before turning the focus on other noteworthy results of the present study.

In line with previous findings [6, 20, 40] we found that systematic observation of facial responses helps to recognize whether an individual is in pain or not, without any additional information about person and context. Therefore, focusing on facial responses to pain is definitely of diagnostic value. One might wonder why not all items of our PAIC-FACE-SCALE (research version) were of help in this respect, given that all items are from established observational scales for pain assessment in dementia [14]. However, only 7 out of 12 facial descriptor items supported the differentiation between no-pain and pain, which were mainly the anatomically orientating items (5 items; e.g. “opened mouth”) and emotionally interpreting items (2 items; e.g. “looking frightened”). Thus, in accordance with previous notions [6], our findings also suggest that several established observational pain assessment scales include items that do not seem to be truly pain-indicative (e.g. “seeming disinterested”, “empty gaze”).

Although these 7 facial descriptor items supported the differentiation of no pain from weak to moderate pain, their power to predict the intensity of self-report of the observed individual was (while significant) only small. Nevertheless, we like to state that these items met our expectations for two reasons. First, the use of these specific facial descriptor items performed much better than unspecific and global evaluations of pain as provided by items such as “how intense is the pain the individual is experiencing?” The fine-grained and standardized look into the face of persons suffering from pain apparently increments diagnostic accuracy. Second, the weak association between facial descriptor items and self-report ratings is in line with established empirical findings that facial expressions and self-report ratings are not closely related, but seem to encode different aspects of the multi-dimensional pain experience [41, 42]. Furthermore, there is evidence [41] that the facial expression of pain is better to reflect within-subject changes in pain intensity (course of pain) than between-subject variations (difference between individuals) as studied in the present study.

Our data suggests that patients with dementia do not lose the capability to broadcast their experience of pain via facial responses. Indeed, the observers in our study were even more successful in differentiating conditions with and without pain when they observed facial responses of patients with dementia compared to healthy elderly persons. This is in line with previous findings that FACS-coded facial responses in patients with dementia are as pain-specific as in healthy controls [8, 11]. This is also true for patients with more advanced stages of dementia who lack self-report ratings [8, 11]. Thus, although we excluded these patients from the present study (given their missing self-report) we are confident that our findings can be generalized to more advanced stages of dementia. The slightly lower correlations between facial descriptor items and the self-report found for patients with dementia are likely due to a worsening of self-report [1] and does not question the intact capacity to express pain via facial responses. There might be subtle differences in the quality of expression because different items proved most predictive for the two groups of observed individuals (for healthy individuals: “opened mouth”, “frowning”; for patients with dementia: “opened mouth”, “raising upper lip”). However, firm conclusions regarding this matter are not yet possible.

Interestingly, we could replicate evidence [20] that hospital and elderly care nurses are - as observers - not better in detecting pain and predicting self-report of pain than laypersons matched in age, gender and education when only using facial responses as diagnostic information. Thus, under the limited conditions of watching only the facial responses of a person in pain, the everyday professional contact with persons in pain and the earlier training as nurses does not make nurses superior as observers. However, this does only mean that nurses cannot derive more pain-indicative information from the facial expression of pain and does not exclude that they can better use other diagnosis-relevant information about patient and context.

What are the consequences of these findings for the further development of the PAIC-FACE-SCALE? The number of items can be reduced to 7 or less, with the notion that the anatomically orientating items are most pain-indicative: “opened mouth”, “raising upper lip”, “frowning” and “narrowing eyes”. This is in line with the findings of Sheu et al. [6], who also found that those scales, which include anatomically descriptive items, showed best reliability and validity. Adding some of the emotionally interpreting items like “looking frightened” or “looking tense” might further increment the diagnostic accuracy. However, further tests on the psychometric properties of the 13 items of the PAIC-FACE-SCALE (e.g. factor analysis) will be necessary for ultimate conclusion, which items can be kept for a final version. The finding, that certain observational items did not help at all to differentiate non-painful from painful conditions and were not useful to predict the self-report of the observed persons, is undoubtedly noteworthy because all items stem from internationally established observational scales for the assessment of pain in dementia. It may well be that other features of these scales compensate for the weak operationalization of facial responses to pain. Furthermore, our tests only scrutinized the capacity of facial descriptor items to indicate acute but not chronic pain. Given that facial responses to pain seem to be similar for acute and chronic pain conditions [36, 37], we expect similar outcomes for chronic pain conditions. Nevertheless, we cannot exclude that some of the non-pain-indicative items in the present study might prove useful in a clinical context.

It is very noteworthy for clinical use that the specific pain-indicative facial items clearly outperformed general evaluations of pain (e.g. “pained expression”). Thus, using observational shortcuts by simply asking the observer to rate their general impression of the patient is not advisable; but fine-grained as well as specific observations are necessary instead.

It is further noteworthy that the best performing anatomically orientating items are labelled almost identical as the so-called Action Units (AU) of the Facial Action Coding System (FACS) [7], which have shown to be pain-indicative over the years. Nevertheless, scoring facial responses in “real-time” with the PAIC-FACE-SCALE is difficult to directly compare to slow-motion (frame-by-frame) FACS coding, as indicated by the weak to moderate correlations between scale ratings and FACS coding. Only the facial descriptor item “opened mouth”, which corresponded with AU 25_26_27, correlated well with the FACS coding. It should be pointed out that these weak to moderate correlations were obtained when the observer could focus all attention on this task, which will rarely be the case in the everyday practice of pain care. This is again a clear indication of how many influences on the observation of even clearly defined behaviors like the facial response to pain exist [43], with method and time for observation being key factors but surely not the only ones.

One might argue that our observers fulfilled their task of rating facial responses under too ideal conditions with stable frontal view on facial activity. For sure, we did not intend to simulate everyday conditions of pain care because we liked to see what the standardized observation of facial responses might achieve under best possible conditions. Thus, we learned from the present study that a lot of effort will be necessary to keep the good but not excellent quality of our observational tool as shown in our experiment also in everyday pain care.

Based on our findings (see regression analyses), we would also suggest for clinical use to keep the graded intensity scaling of facial descriptors instead of using dichotomous yes/no answers. Especially with regard to items like “frowning” or “opened mouth”, it seems advisable to differentiate between the intensities of these facial responses.

## Conclusions

In summary, facial descriptor items guided the observation of healthy elderly and patients with dementia by geriatric nurses and laypersons, so that acute experimental pain became detectable by only monitoring the facial responses of the observed individual. The best facial descriptor items, which were mainly anatomically-oriented, also allowed for significantly predicting the self-report of the observed people and were better in this respect than global pain evaluations. In line with previous findings however, the observation of facial responses were only weakly correlated with self-report, showing that the observation of facial responses is not a complete substitute of self-report but an additional information about the pain status. There was no indication that the observation of facial responses in patients with dementia leads to less valid findings than in healthy individuals. Thus, the PAIC-FACE-SCALE as the source of the verbal descriptor items under study, which were all taken from internationally established observational scales, promised to become a brief (after item reduction) and valid tool for assessment of pain in dementia focusing on the facial responses to pain; the combination with observational (sub)-scales covering other behavioral domains (body posture/movement, vocalization) will follow.

## Footnotes

1 PAIC stands for Pain And Impaired Cognition
